# Prevalence, awareness, treatment and control of dyslipidemia in older persons in urban and rural population in the Astana region, Kazakhstan

**DOI:** 10.1186/s12889-017-4629-5

**Published:** 2017-08-11

**Authors:** Adil Supiyev, Talgat Nurgozhin, Zhaxybay Zhumadilov, Anne Peasey, Jaroslav A. Hubacek, Martin Bobak

**Affiliations:** 1 Department of Epidemiology and Public Health, University College London, 1–19 Torrington Place, London, WC1E 6BT UK; 2 Laboratory of Epidemiology and Public Health, Center for Life Sciences, PI National Laboratory Astana, Nazarbayev University, Kabanbay Batyr Ave. 53, Astana, Kazakhstan 010000; 3 Centre for Experimental Medicine, Institute of Clinical and Experimental Medicine, Videnska 1958/9, 14021 Prague 4, Czech Republic

**Keywords:** Hypercholesterolemia, Dyslipidemia, Socioeconomic factors, Central Asian countries, Kazakhstan

## Abstract

**Background:**

Despite high cardiovascular mortality in Central Asian republics of the former Soviet Union, there is limited information about major risk factors, including blood lipids. We investigated the prevalence of impaired concentrations of blood lipids, the awareness, treatment and control of hypercholesterolemia, and factors associated with these indicators in urban and rural populations in Kazakhstan.

**Methods:**

We conducted a cross-sectional study of random urban and rural population samples (the state capital Astana and Akmol village). Men and women aged 50–74 years were examined; a total of 954 adults participated (response rate 59%). Serum concentrations of total, LDL and HDL cholesterol and triglycerides and a range of other cardiovascular risk factors were measured.

**Results:**

The overall prevalence of hypercholesterolemia (total cholesterol ≥6.2 mmol/l) was 37%; among subjects with hypercholesterolemia, 57% were aware of their condition, 41% took medication and 23% had total cholesterol <6.2 mmol/l (4.5% <5 mmol/l). The prevalence, awareness, treatment, and control of hypercholesterolemia were all higher in the urban than the rural area. Similarly, the proportions of subjects with impaired concentrations of specific lipids fractions were also considerably higher in the urban population. Most associations with other covariates were in the expected direction.

**Conclusions:**

This study found relatively high prevalence of dyslipidemia in the Kazakh population, and the blood lipid profile was less favourable in the urban area. These pronounced urban–rural differences may be related to urbanization, the associated nutrition transition and to access to health care.

**Electronic supplementary material:**

The online version of this article (doi:10.1186/s12889-017-4629-5) contains supplementary material, which is available to authorized users.

## Background

Dyslipidemia is an important modifiable risk factor for cardiovascular disease (CVD); based on WHO global estimates, dyslipidemias cause one third of ischemic heart disease and one fifth of global cerebrovascular disease, and equates to nearly 2.6 million deaths every year worldwide [1]. Various lipid abnormalities, such as increased total and low-density lipoprotein cholesterol, low concentrations of high-density lipoprotein cholesterol and high triglycerides concentrations, and their combinations, have been implicated as potential independent predictors of CVD [2–4].

According to WHO, the age-standardized prevalence of raised total cholesterol (≥6.2 mmol/l) in Kazakhstan was estimated as 12%, which is similar to Russian Federation (15%) but considerably higher than in other Central Asian countries. The highest estimate is for Turkmenistan with 8% and the lowest in Tajikistan with 5%, although estimates in Central Asian countries are much lower than developed countries (e.g. 25% in Germany and 22% in Great Britain) [5]. Unfortunately, as there have been no representative individual-level studies on dyslipidemia in the Central Asian region, it is hard to reliably assess the magnitude of the problem. Given the rapid societal and economic changes after the breakup of the former Soviet Union and the associated increase in CVD mortality rates, assessment of dyslipidemias and other cardio-metabolic factors in the Central Asian republics is an urgent public health matter. The traditional Kazakh diet included high consumption energy-dense food (particularly saturated fat) but it was it was combined with high physical activity. By contrast, the post-Soviet transition period has been characterised by rapid westernisation and urbanisation, leading to an increase in food availability, including an increasing density of food and wine stores, in this formerly isolated society. This resulted in changing patterns of food consumption at the time of declining physical activity [6, 7].

The aim of the present study was to investigate the mean levels and prevalence of impaired concentrations of serum lipids, and to present estimates of prevalence, awareness, treatment, and control levels of hypercholesterolemia in general population samples in an urban area (Astana, the capital city) and an adjacent rural area (Akmol village) in Kazakhstan.

## Methods

### Study population

This cross-sectional study was implemented in the Astana region between November 2012 and March 2015. The urban area was Astana city, the capital of Kazakhstan, and the rural population was the Akmol village. Astana, the capital of Kazakhstan, is important because it sets the trend for the rest of the country. It is likely that the current situation of Astana is the future of other urban settings in Kazakhstan and other Central Asian cities. The rural population around Astana is large and consists of several villages. The Akmol village, located approximately 50 km from Astana city, was selected because it is sufficiently far from Astana city to preserve its rural nature while it is still close enough to allow regular transport of biological samples. The participants were randomly selected (stratified by sex and 5-year age groups) from the lists of all residents in the age range 50–74 years who were registered at local outpatient clinics (polyclinics). The recruiting procedures included invitation to a polyclinic by landline phone calls; if telephone communication was not successful, participants were visited at their home and invited to participate in the study. A total of 954 of adults aged 50–74 years were recruited (480 in Astana city and 474 in Akmol). The overall response rate was 59% (56% in urban and 63% in rural area).

Data were collected by trained doctors using a standardized survey protocol and paper questionnaires; the data were subsequently entered into a database. The structured questionnaire included an overall assessment of the patient’s health, medical history, lifestyle and socio-economic indicators. The examination included blood pressure measurement, anthropometric measures (height, weight, waist and hip circumference) and collection of a venous blood sample.

## Measurements

According to the previously published analyses on diabetes and arterial hypertension [8, 9] the subjects were invited to visit the polyclinic early in the morning after an overnight fast. The fasting status was self-reported by participants and recorded in the questionnaire. Those who did not meet the fasting status requirements (fasting for at least 12 h prior to examination) were invited to visit the polyclinic on another occasion. In both Astana city and Akmol village, all blood samples were collected in EDTA vacutainer. Vacutainers were carefully shaken, and then centrifuged. Cooled serum samples were transferred to Astana city, and serum total cholesterol (TC), low-density lipoprotein cholesterol (LDL-C), high-density lipoprotein cholesterol (HDL-C), triglycerides (TG) and glucose concentration were measured by automatic modular analyzer Cobas 8000, Roche Diagnostics (Germany) using the following reagents for the assays (reagent catalog number: 05168538190 (TC), 05171369190 (LDL-C), 05168805190 (HDL-C), 05171407190 (TG)). The maximum delay time for this biochemical analysis was 4 h after blood collection.

### Hypercholesterolemia and abnormal lipid concentrations

To study prevalence, awareness, treatment and control of hypercholesterolemia, we used two conventional cut-offs of TC concentrations. First, the primary definition of hypercholesterolemia was set as total serum cholesterol ≥6.2 mmol/l (240 mg/dl) and/or current use of cholesterol lowering medication [10]. Secondly, for comparisons with other studies, we used another cut-off point for hypercholesterolemia total cholesterol ≥5.0 mmol/l and/or current use of cholesterol lowering medication [11]. Awareness status was assessed by the question whether the subject had been told by a doctor that they had high level of cholesterol lipids. Because the vast majority of the general public do not know about specific lipid fractions, we asked about total cholesterol levels in this question, similar to many other studies, e.g. the WHO MONICA Project [12]. Subjects who reported taking regular cholesterol lowering medications were considered to be on treatment for high cholesterol. Control of hypercholesterolemia among those with high cholesterol was defined as fasting serum total cholesterol less than 6.2 mmol/l (or 5.0 mmol/l in secondary analyses).

For other lipids, we used the cut-offs recommended by the National Cholesterol Education Program Expert Panel on Detection, Evaluation, and Treatment of High Blood Cholesterol in Adults (ATP III): raised level of low-density lipoprotein cholesterol ≥4.15 mmol/l (160 mg/dl), raised serum triglycerides levels ≥2.26 mmol/l (200 mg/dl) and low level of high-density lipoprotein cholesterol was set as <1.04 mmol/l (40 mg/dl) for men and for women [10].

### Obesity

We used two indices of obesity: body mass index (BMI, kg/m^2^) and waist to hip ratio (WHR) to assess overall and central obesity, respectively. Weight of subjects with light clothing was measured by standard balanced scales to the nearest 0.1 kg, and height was measured by a steel stadiometer to the nearest 0.1 cm. As recommended by WHO, BMI was classified as normal (BMI 18.5–24.9), overweight (BMI 25–29.9) and obese (BMI over 30 kg/m^2^). Waist and hip circumferences were measured using a standard tape measure. The waist circumference was measured halfway between the costal margin and iliac crest, and hip circumference was measured at the widest portion of the buttocks. WHR was categorized into two groups based on WHO cut-off points: central obesity was defined as a waist to hip ratio above 0.90 for men and above 0.85 for women [13].

### Diabetes

The diabetes was classified as fasting plasma glucose (FPG) concentration more than or equal to 7.0 mmol/l (126 mg/dl) or self-reported diabetes medication use; the questionnaire did not specify the type of diabetes (I or II). The measurements and derived indices were described in detail previously [9].

### Arterial hypertension

Before blood pressure measurement participants were asked to sit calmly for 5 min. Blood pressure was measured by OMRON M6 comfort with three consecutive readings on the right arm in sitting position, with a two-minute interval between measurements. Hypertension was defined as SBP ≥140 mmHg and/or DBP ≥90 mmHg, and/or the use of antihypertensive medication in the last 2 weeks. More details about the procedures of blood pressure measurements were reported elsewhere [8].

### Socio-demographic and behaviour characteristics

The socio-demographic and behaviour characteristics, as previously reported [8, 9] included age, sex and urban vs. rural area of residence and several socioeconomic and demographic variables. Marital status was classified as married or unmarried (widowed, divorced and single) and education was divided into primary or less, vocational/secondary, and university. The ethnicity was classified as Kazakh, Russian and other. Car ownership (yes vs. no) and material deprivation were used as markers of the economic status. Material deprivation was assessed by three questions (how often participants do not have enough money for food, clothes and paying bills for their households), and participants were classified into three categories (high, intermediate and low). Smoking status was evaluated using two questions: if subject currently smokes, and if not, did he/her smoke in the past, and accordingly individuals were categorized as current smokers, ex-smokers, or non-smokers.

### Statistical analysis

In descriptive tables we present unadjusted frequencies of all predictor and outcome variables by urban or rural residence. The association between outcomes and covariates (socio-demographic characteristics, BMI, WHR, behaviours, hypertension and diabetes) was estimated using logistic regression adjusted for age and sex. To estimate independent effects of each covariate on the prevalence of hypercholesterolemia, a multivariable model was built with all covariates entered in the logistic regression model. As the numbers of subjects in models analysing awareness, treatment and control of hypercholesterolemia were too small for meaningful multivariable analyses, only age-sex adjusted estimates are reported for these outcomes. For supplementary tables, we also estimated the age-sex-adjusted means of all serum lipids by all covariates, using linear regression. To account for intra-cluster correlation (within each area), we initially used the “cluster” option in STATA; however, since this approach yielded very small standard errors (possibly due to negative intra-cluster correlation), we used robust regression (option “robust” in STATA) which provided more conservative estimates. All analyses were performed using STATA software, version 12 (College Station, Texas, USA).

## Results

Table 1 shows descriptive characteristics of the urban and rural population samples in the Astana region in Kazakhstan. The mean age of the participants was almost similar between the urban and rural regions. Among cardiometabolic factors, 13% of subjects had diabetes, 72% had hypertension and 44% were obese (there were no underweight individuals in the study), and 72% had central obesity. BMI, waist-hip ratio, diabetes and fasting serum lipids, except HDL-C were higher in urban than rural area. The percentage of smokers, higher education and car ownership were also higher in urban than rural area.

**Table 1:** Descriptive characteristics of the study population in Astana region, Kazakhstan

Variable name	Astana city (urban)	Akmol village (rural)	Both
Total number of participants	*N* = 480	*N* = 474	*N* = 954
Sex, (%)			
Males	45.8%	41.4%	43.6%
Females	54.2%	58.7%	56.4%
Age mean, (SD)	61.2 (7.3)	60.3 (7.3)	60.7 (7.3)
Total cholesterol (mmol/l), mean (SD)	5.89 (1.51)	5.44 (1.09)	5.67 (1.34)
LDL-C (mmol/l), mean (SD)	3.71 (0.97)	3.52 (0.96)	3.62 (0.97)
HDL-C (mmol/l), mean (SD)	1.30 (0.36)	1.46 (0.40)	1.38 (0.39)
TG (mmol/l), mean (SD)	1.66 (1.08)	1.52 (1.04)	1.59 (1.06)
Diabetes prevalence, (%)	16.3%	8.7%	12.5%
Arterial Hypertension prevalence, (%)	70.3%	74.1%	72.2%
BMI, mean (SD)	29.8 (4.9)	29.2 (5.3)	29.5 (5.1)
BMI categories, (%)			
18.50 - 24.99 (normal)	13.4%	20.3%	16.8%
25.00 - 29.99 (pre-obese)	40.6%	37.9%	39.3%
Over 30 (obese)	46.0%	41.7%	43.9%
Waist–hip ratio, (%) ≥0.9 in men and ≥0.85 in women	81.7%	61.7%	71.7%
Smoking status			
Current smoker	17.4%	13.9%	15.6%
Ex-smoker	21.6%	16.5%	19.0%
Non-smoker	61.0%	69.7%	65.3%
Marital status, (%)			
Married	73.1%	73.7%	73.4%
Unmarried	26.9%	26.3%	26.6%
Education, (%)			
1 Primary	36.3%	39.2%	37.7%
2 Secondary	21.3%	45.3%	33.2%
3 Higher	42.5%	15.5%	29.2%
Car ownership, (%)			
No	38.8%	50.9%	44.8%
Yes	61.2%	49.1%	55.2%
Ethnicity, (%)			
Kazakhs	57.5%	58.4%	58.0%
Russians	26.0%	27.2%	26.6%
Others	16.5%	14.4%	15.4%
Deprivation level, (%)			
high level of deprivation	26.9%	26.2%	26.6%
Intermediate level of deprivation	29.4%	33.8%	31.6%
low level of deprivation	43.6%	40.0%	41.8%

Table 2 presents the prevalence, awareness, treatment and control of hypercholesterolemia. In the total sample of 954 subjects, 37.2% were classified as having hypercholesterolemia; the proportion of subjects with hypercholesterolemia was almost twice as high in the urban compared to rural area. Among subjects with hypercholesterolemia, 56.6% were aware of their condition, 40.6% took medication, and 23.4% had total cholesterol concentrations <6.2 mmol/l. Among those aware of the condition, 70.7% were taking medication, and among those taking medication, 57.6% had concentrations <6.2 mmol/l. There were pronounced differences between urban and rural areas. The prevalence of awareness, treatment and control of hypercholesterolemia were about 1.5 times higher in the urban vs. rural area. Prevalence, awareness and treatment were about 1.5 times higher in women than men in both urban and rural areas, although control was better among men than women in both areas.

**Table 2:** Prevalence, awareness, treatment and control of hypercholesterolemia (HC) in Astana region, Kazakhstan (total cholesterol ≥6.2 mmol/l)

	Men (*n* = 416)			Women (*n* = 538)			Both sexes (*n* = 954)		
	Prevalence	95% CI	Cases/All	Prevalence	95% CI	Cases/All	Prevalence	95% CI	Cases/All
Combined Astana city and Akmol village									
Prevalence of HC among all	28.4%	24.0–32.7	118/416	44.1%	39.8–48.3	237/538	37.2%	34.1–40.3	355/954
Awareness among all cases of HC	47.5%	38.3–56.6	56/118	61.2%	54.9–67.4	145/237	56.6%	51.4–61.8	201/355
Treatment among all cases of HC	33.1%	24.4–41.7	39/118	44.3%	37.9–50.7	105/237	40.6%	35.4–45.7	144/355
Treatment among aware	67.9%	55.2–80.5	38/56	71.7%	64.3–79.1	104/145	70.7%	64.3–77.0	142/201
Control among all cases of HC	26.3%	18.2–34.3	31/118	21.9%	16.6–27.2	52/237	23.4%	19.0–27.8	83/355
Control among treated	79.5%	66.2–92.7	31/39	49.5%	39.8–59.2	52/105	57.6%	49.5–65.8	83/144
Astana city (urban, *n* = 480)									
Prevalence of HC among all	38.2%	31.7–44.7	84/220	57.3%	51.3–63.4	149/260	48.5%	44.1–53.0	233/480
Awareness among all cases of HC	56.0%	45.1–66.8	47/84	72.5%	65.2–79.7	108/149	66.5%	60.4–72.6	155/233
Treatment among all cases of HC	36.9%	26.4–47.4	31/84	49.7%	41.5–57.8	74/149	45.1%	38.6–51.5	105/233
Treatment among aware	63.8%	49.6–78.1	30/47	68.5%	59.6–77.4	74/108	67.1%	59.6–74.6	104/155
Control among all cases of HC	29.8%	19.8–39.7	25/84	24.8%	17.8–31.9	37/149	26.6%	20.9–32.3	62/233
Control among treated	80.7%	65.9–95.4	25/31	50.0%	38.3–61.7	37/74	59.1%	49.5–68.6	62/105
Akmol village (rural, *n* = 474)									
Prevalence of HC among all	17.4%	12.0–22.7	34/196	31.7%	26.2–37.2	88/278	25.7%	21.8–29.7	122/474
Awareness among all cases of HC	26.5%	10.8–42.1	9/34	42.1%	31.5–52.6	37/88	37.7%	29.0–46.4	46/122
Treatment among all cases of HC	23.5%	8.5–38.6	8/34	35.2%	25.0–45.4	31/88	32.0%	23.6–40.4	39/122
Treatment among aware	88.9%	63.3–114.5	8/9	81.1%	67.8–94.3	30/37	82.6%	71.2–94.0	38/46
Control among all cases of HC	17.7%	4.1–31.1	6/34	17.1%	9.0–25.1	15/88	17.2%	10.4–24.0	21/122
Control among treated	75.0%	36.3–113.7	6/8	48.4%	29.8–67.0	15/31	53.9%	37.5–70.2	21/39

The prevalence, awareness, treatment and control of hypercholesterolemia using the lower cut-off of ≥5.0 mmol/l are shown in Additional file 1: Table S1. Using this lower cut-off, the overall prevalence of hypercholesterolemia was 72.8%. There is consistent distribution of all characteristics, with higher levels in urban area. However, the gap between urban and rural areas increased considerably; the prevalence of awareness and treatment of hypercholesterolemia were more than two times higher in urban area. Successful control of hypercholesterolemia in the rural area was 1.3% compared with 7% in Astana city; among those aware of having hypercholesterolemia, the proportions with successful control was 10.3 and 25.7%, respectively. Differences between men and women were similar to those presented for the standard hypercholesterolemia (≥6.2 mmol/l).

Age and sex standardized means of serum lipids and respective age and sex adjusted odds ratios for impaired lipid concentrations by covariates are shown in Additional file 2: Table S2. Impaired lipid concentrations were more common in women, those who were obese and in subjects with diabetes and hypertension. The odds of dyslipidemia in the rural area was about half of that in Astana city. Car ownership, deprivation levels and education were statistically significantly associated with dyslipidemia prevalence. Russian ethnicity was associated with high rates of impaired lipid concentrations.

Table 3 shows the odds ratios of hypercholesterolemia prevalence, awareness, treatment and control by covariates. In age-adjusted model, the prevalence of hypercholesterolemia was associated with being female, higher age, obesity and central obesity, diabetes and hypertension. The odds of prevalence, awareness, treatment and control were also substantially lower in the rural vs. urban area. Among the socio-demographic measures, deprivation levels and education were statistically significantly associated with (increased) hypercholesterolemia prevalence, awareness and control. As mentioned earlier, Russian ethnicity had increased prevalence of hypercholesterolemia. Multivariable analysis of hypercholesterolemia prevalence (Table 3, column 3) largely confirmed these findings, except that the association with BMI, central obesity and diabetes were reduced, mainly because these variables were strongly mutually correlated.

**Table 3:** Association of selected factors with the prevalence, awareness, treatment and control of HC (≥6.2 mmol/l) in in Astana region, Kazakhstan, OR (95% CI)

	Prevalence^a^ OR (95% CI)	Prevalence^b^ OR (95% CI)	Awareness^a,c^ OR (95% CI)	Treatment^a,c^ OR (95% CI)	Control^ac^ OR (95% CI)
Sex					
Males	1	1	1	1	1
Females	1.98 (1.50–2.60)	2.54 (1.63–3.96)	1.75 (1.11–2.74)	1.60 (1.00–2.55)	0.78 (0.47–1.31)
Age groups (years)					
50-54	1	1	1	1	1
55-59	1.55 (1.06–2.26)	1.63 (1.07–2.49)	1.32 (0.72–2.43)	1.48 (0.79–2.77)	0.98 (0.47–2.04)
60-64	1.44 (0.97–2.14)	1.28 (0.83–1.99)	1.20 (0.64–2.28)	1.55 (0.81–2.98)	1.25 (0.59–2.65)
65-69	1.32 (0.86–2.05)	1.20 (0.74–1.95)	1.45 (0.71–2.95)	1.29 (0.62–2.65)	1.47 (0.66–3.28)
70-75	1.44 (0.93–2.24)	1.01 (0.61–1.70)	1.16 (0.57–2.35)	1.24 (0.60–2.56)	1.23 (0.54–2.82)
Urban/Rural					
Astana (urban)	1	1	1	1	1
Akmol (rural)	0.34 (0.26–0.45)	0.37 (0.26–0.51)	0.27 (0.17–0.43)	0.53 (0.33–0.85)	0.59 (0.34–1.03)
BMI (kg/m2)					
18.5-24.9	1	1	1	1	1
25-29.9	1.48 (0.98–2.24)	1.00 (0.62–1.61)	1.02 (0.51–2.06)	0.62 (0.31–1.27)	0.94 (0.41–2.14)
≥ 30	1.77 (1.18–2.66)	1.12 (0.68–1.82)	1.68 (0.85–3.31)	0.91 (0.46–1.79)	1.08 (0.49–2.40)
WHR, obesity					
No	1	1	1	1	1
Yes	2.12 (1.53–2.92)	1.55 (1.06–2.27)	1.30 (0.76–2.22)	1.27 (0.73–2.19)	0.69 (0.37–1.27)
Diabetes					
No	1	1	1	1	1
Yes	1.71 (1.15–2.54)	1.25 (0.81–1.94)	2.29 (1.22–4.27)	1.83 (1.02–3.26)	2.00 (1.08–3.72)
Smoking					
Current Smoker	1	1	1	1	1
Ex-smoker	1.05 (0.65–1.69)	0.98 (0.58–1.65)	2.28 (1.02–5.11)	1.70 (0.74–3.90)	1.01 (0.40–2.53)
Non-smoker	0.74 (0.46–1.17)	0.83 (0.50–1.39)	1.25 (0.60–2.63)	1.10 (0.51–2.37)	1.29 (0.54–3.07)
Hypertension					
No	1	1	1	1	1
Yes	1.44 (1.05–1.97)	1.50 (1.05–2.13)	1.28 (0.77–2.15)	1.35 (0.79–2.29)	1.52 (0.80–2.91)
Marital status					
Unmarried	1	1	1	1	1
Married	0.89 (0.64–1.22)	0.85 (0.59–1.24)	0.98 (0.59–1.63)	1.07 (0.65–1.77)	1.82 (0.97–3.39)
Education					
Primary	1	1	1	1	1
Vocational	0.81 (0.58–1.12)	0.97 (0.67–1.39)	0.92 (0.54–1.56)	1.13 (0.66–1.95)	1.14 (0.62–2.12)
Higher	1.37 (0.98–1.90)	1.06 (0.73–1.53)	1.95 (1.15–3.33)	1.65 (0.98–2.78)	1.10 (0.60–2.00)
Ethnicity					
Kazakh	1	1	1	1	1
Russian	1.39 (1.01–1.90)	1.33 (0.93–1.90)	0.80 (0.49–1.32)	1.05 (0.64–1.73)	0.95 (0.54–1.68)
Other	1.44 (0.99–2.11)	1.36 (0.90–2.07)	0.76 (0.42–1.37)	1.09 (0.60–1.98)	0.72 (0.35–1.50)
Car ownership					
No	1	1	1	1	1
Yes	1.22 (0.92-1.62)	1.06 (0.77-1.46)	1.25 (0.79-1.98)	0.87 (0.55-1.37)	0.84 (0.49-1.44)
Deprivation					
High level	1	1	1	1	1
Intermediate	1.35 (0.94–1.94)	1.68 (1.12–2.52)	1.20 (0.67–2.14)	1.36 (0.75–2.44)	1.97 (0.95–4.11)
Low level	1.50 (1.07–2.11)	1.73 (1.18–2.54)	1.20 (0.69–2.07)	1.14 (0.65–1.99)	1.80 (0.89–3.63)

^a^age and sex adjusted, ^b^fully adjusted for all variables in the table; ^c^Among those with hypercholesterolemia

## Discussion

In this population-based study in the Astana region of Kazakhstan, we found an overall prevalence of hypercholesterolemia of 37.2% using the ≥6.2 mmol/l cut-off and 72.8% using the 5 mmol/l cut-off. There were large differences between urban and rural settings, with almost all lipid indicators being less favourable among urban residents. Most associations of hypercholesterolemia with covariates were in the expected direction; although we were surprised to find higher prevalence of lipid abnormalities among ethnic Russians and among more affluent groups.

Our study has several limitations. Firstly, the cross-sectional study design cannot establish the direction of some associations. Education is generally completed in early life and is unlikely to be modified by health outcomes, but we are less sure about deprivation, car ownership and health behaviours. Second, the response rate of the study was modest, however a response rate of 60% is similar to many contemporary studies in Europe and elsewhere [14], but as responders in epidemiological studies have higher socio-economic status and better health than non-responders [15] we also cannot discount the possibility that a study based in out-patient department (polyclinic) may have attracted less healthy and more aware population to participate in this screening project. Therefore, the moderate response rates may lead to both under- and overestimation of dyslipidemia prevalence. The response rates were slightly higher in Akmol area, but the difference was not sufficient to introduce selection bias.

Third, adjustment for potential confounders may be incomplete; in particularly, urban and rural areas may differ in many aspects potentially associated with blood lipids, and we were unable to adjust for all of these factors (e.g. nutrition).

The rural and urban areas in this study were intentionally selected to assess urban–rural differences. Astana is a very young capital city with a high proportion of civil servants and more affluent and relatively better-educated population. Akmol is a rural area proximal to Astana but it is remote enough to have a stable population with low level of migration. Nevertheless, it is likely that more distant areas, or other regions in Kazakhstan, would present even larger differences in socio-demographic and health indicators compared to Astana city. The urban–rural differences in dyslipidemias are consistent with distribution of other variables, such as obesity or diabetes. The validity of our findings is supported by the fact that being female, higher BMI and WHR, diabetes and hypertension were also positively significantly associated with dyslipidemia, similarly to other published studies [16, 17].

Our literature review did not identify any previously published studies on the awareness, treatment and control of hypercholesterolemia or determinants of any other lipid abnormalities in Kazakhstan and other Central Asian countries. Despite the strong association of dyslipidemia with cardiovascular risk, with a few exceptions [18] there is a striking lack of studies of this important aspect of cardiovascular health in the Central Asian region. The available information is largely limited to WHO reports on cardiovascular disease risk factors, with the source of the data for this region sometimes uncertain and or being based on extrapolations from other regions [19, 20].

The overall high levels of awareness, treatment and control in this study could be attributed to wealthier and better educated population in Astana city. The high prevalence of lipid abnormalities, as well as high levels of dyslipidemias, diabetes and obesity in Astana city may point towards large-scale globalization and urbanization processes, with the associated the nutrition transition with easy access to fast food industry and sedentary lifestyle. This is consistent with reported findings from other developing Asian populations with higher rates of various health outcomes of non-communicable diseases in urban compared to rural areas [21].

Studies in low and middle-income countries frequently reported high prevalence of dyslipidemia in urban areas and low prevalence of awareness, treatment and control levels in rural settings [22, 23]. We found large disparities in hypercholesterolemia prevalence between the two locations. All hypercholesterolemia indices were lower in Akmol village than in Astana. However, using lower TC cut-off point (≥5.0 mmol/l) revealed an even more marked difference between urban and rural populations regarding awareness and treatment of disease, and an extremely low proportion of controlled cases of hypercholesterolemia in the rural area.

Several recent studies have examined the distribution of dyslipidemia. In China, the prevalence of dyslipidemias was much lower compared to our study (12% had TC ≥6.2 mmol/l, 18% had high LDL-C, 12% had low HDL-C and 15% had high TG); similarly, the levels of awareness, treatment and control indices (22, 10 and 4% respectively) were much lower. On the other hand, the Chinese study population included younger subjects (18–79 years) [24]. A Turkish study of 20–83 year olds showed much higher prevalence of dyslipidemias (43% had TC ≥5.0 mmol/l, 42% had low HDL-C, 36% had high LDL-C, and 36% had high TG) [25]. Compared to similar aged population groups, our results were similar to Italy with 78.2% of subjects with hypercholesterolemia (≥5.0 mmol/l) [26]. Internationally, lipid abnormalities tend to be more common in more affluent countries, [27] and this pattern may be analogous to our finding of higher prevalence of dyslipidemia in the urban vs. rural populations.

Our findings confirm the association of lipid abnormalities with the cardiometabolic risk factors, obesity, diabetes and hypertension [28]. Behavioural differences, including smoking, were also reported to be associated with hypercholesterolemia and other dyslipidemias [29, 30]. We found only small differences in abnormal lipid prevalence by education and car ownership, and deprivation levels were inversely associated with increased odds of dyslipidemias. The higher prevalence of dyslipidemia in ethnic Russians in Kazakhstan in this study may reflect differences in lifestyle and particularly in nutrition [31]; however, in absence of reliable individual-level data on nutrition this is only a speculation.

## Conclusions

This study found relatively high prevalence of dyslipidemia in the Kazakhstan population, and the lipid profile was much less favourable in the urban area, while awareness and treatment of dyslipidemia was less common in rural areas and persons with lower education. Providing such estimates is a necessary first step in addressing the problem. The pronounced urban vs. rural differences raise questions about both the potential distal and proximal causes of dyslipidemias in this middle-income setting which should be addressed by future research. The results suggest that health services access and appropriate interventions should be improved in rural areas and for persons with lower socioeconomic status.

## Additional files

Additional file 1: Table S1. Prevalence, awareness, treatment and control of hypercholesterolemia (HC) in Astana region, Kazakhstan (total cholesterol ≥5 mmol/l). (DOC 49 kb)

Additional file 2: Table S2. Factors associated with serum cholesterols in Astana region, Kazakhstan, OR (95% CI). (DOC 92 kb)
