# Supplementary material for: Prevalence, awareness, treatment and control of dyslipidemia in older persons in urban and rural population in the Astana region, Kazakhstan
Source: BMC Public Health. 2017 Aug 11;17:651. doi: 10.1186/s12889-017-4629-5 (PMC5553733; doi:10.1186/s12889-017-4629-5)
Supplement: Supplementary file 1 — Prevalence, awareness, treatment and control of hypercholesterolemia (HC) in Astana region, Kazakhstan (total cholesterol ≥5 mmol/l). (DOC 49 kb) [file 12889_2017_4629_MOESM1_ESM.doc]

**Additional file 1: Table S1. Prevalence, awareness, treatment and control of hypercholesterolemia (HC) in Astana region, Kazakhstan (total cholesterol ≥ 5 mmol/l)**

|  | **Men (n=416)** | | | **Women (n=538)** | | | **Both sexes (n=954)** | | |
| --- | --- | --- | --- | --- | --- | --- | --- | --- | --- |
|  | *Prevalence* | *95% CI* | *Cases/All* | *Prevalence* | *95% CI* | *Cases/All* | *Prevalence* | *95% CI* | *Cases/All* |
| **Combined Astana city and Akmol village** | | | | | | | | | |
| Prevalence of HC among all | 65.9% | 61.3-70.4 | 274/416 | 78.1% | 74.6-81.6 | 420/538 | 72.8% | 69.9-75.6 | 694/954 |
| Awareness among all cases of HC | 29.9% | 24.5-35.4 | 82/274 | 44.3% | 39.5-49.1 | 186/420 | 38.6% | 35.0-42.2 | 268/694 |
| Treatment among all cases of HC | 14.2% | 10.1-18.4 | 39/274 | 25.0% | 20.8-29.2 | 105/420 | 20.8% | 17.7-23.8 | 144/694 |
| Treatment among aware | 46.3% | 35.3-57.4 | 38/82 | 55.9% | 48.7-63.1 | 104/186 | 53.0% | 47.0-59.0 | 142/268 |
| Control among all cases of HC | 4.7% | 2.2-7.3 | 13/274 | 4.3% | 2.3-6.2 | 18/420 | 4.5% | 2.9-6.0 | 31/694 |
| Control among treated | 33.3% | 17.9-48.8 | 13/39 | 17.1% | 9.8-24.5 | 18/105 | 21.5% | 14.7-28.3 | 31/144 |
| Astana city (urban, n=480) | | | | | | | | | |
| Prevalence of HC among all | 75.5% | 69.7-81.2 | 166/220 | 84.6% | 80.2-89.0 | 220/260 | 80.4% | 76.9-84.0 | 386/480 |
| Awareness among all cases of HC | 36.8% | 29.3-44.2 | 61/166 | 61.8% | 55.3-68.3 | 136/220 | 51.0% | 46.0-60.5 | 197/386 |
| Treatment among all cases of HC | 18.7% | 12.7-24.7 | 31/166 | 33.6% | 27.3-39.9 | 74/220 | 27.2% | 22.7-31.7 | 105/386 |
| Treatment among aware | 49.2% | 36.3-62.1 | 30/61 | 54.4% | 45.9-62.9 | 74/136 | 52.8% | 45.8-59.8 | 104/197 |
| Control among all cases of HC | 7.2% | 3.2-11.2 | 12/166 | 6.8% | 3.5-10.2 | 15/220 | 7.0% | 4.4-9.6 | 27/386 |
| Control among treated | 38.7% | 20.5-56.9 | 12/31 | 20.3% | 10.9-29.6 | 15/74 | 25.7% | 17.2-34.2 | 277/105 |
| Akmol village (rural, n=474) | | | | | | | | | |
| Prevalence of HC among all | 55.1% | 48.1-62.1 | 108/196 | 71.9% | 66.6-77.3 | 200/278 | 65.0% | 60.7-69.3 | 308/474 |
| Awareness among all cases of HC | 19.4% | 11.9-27.0 | 21/108 | 25.0% | 18.9-31.1 | 50/200 | 23.1% | 18.3-27.8 | 71/308 |
| Treatment among all cases of HC | 7.4% | 2.4-12.4 | 8/108 | 15.5% | 10.4-20.6 | 31/200 | 12.7 | 8.9-16.4 | 39/308 |
| Treatment among aware | 38.1% | 15.4-60.7 | 8/21 | 60.0% | 45.9-74.1 | 30/50 | 53.5% | 41.6-65.4 | 38/71 |
| Control among all cases of HC | 0.9% | 0-2.8 | 1/108 | 1.5% | 0-3.2 | 3/200 | 1.3% | 0-2.6 | 4/308 |
| Control among treated | 12.5% | 0-42.1 | 1/8 | 9.7% | 0-20.7 | 3/31 | 10.3% | 0.3-20.2 | 4/39 |
