# Supplementary material for: Prevalence, awareness, treatment and control of dyslipidemia in older persons in urban and rural population in the Astana region, Kazakhstan
Source: BMC Public Health. 2017 Aug 11;17:651. doi: 10.1186/s12889-017-4629-5 (PMC5553733; doi:10.1186/s12889-017-4629-5)
Supplement: Supplementary file 2 — Factors associated with serum cholesterols in Astana region, Kazakhstan, OR (95% CI). (DOC 92 kb) [file 12889_2017_4629_MOESM2_ESM.doc]

**Additional file 2: Table S2. Factors associated with serum cholesterols in Astana region, Kazakhstan, OR (95% CI)**

|  | TC Mean (SE)* | Raised TCPrevalence^1^OR (95% CI) | LDL mean (SE)* | Raised LDLPrevalence^1^OR (95% CI) | HDL mean(SE)* | Low HDLPrevalence^1^OR (95% CI) | TG mean(SE)* | Raised TGPrevalence^1^OR (95% CI) |
| --- | --- | --- | --- | --- | --- | --- | --- | --- |
| **Sex** |  |  |  |  |  |  |  |  |
| Males | 5.12 (0.04) | 1 | 3.47 (0.05) | 1 | 1.29 (0.02) | 1 | 1.67 (0.07) | 1 |
| Females | 5.70 (0.05) | 1.84 (1.38-2.46) | 3.73 (0.04) | 1.82 (1.35-2.45) | 1.45 (0.02) | 0.30 (0.21-0.42) | 1.54 (0.04) | 0.83 (0.59-1.18) |
| P value | <0.001 |  | <0.001 |  | <0.001 |  | 0.093 |  |
| **Age groups (years)** |  |  |  |  |  |  |  |  |
| 50-54 | 5.36 (0.06) | 1 | 3.57 (0.07) | 1 | 1.36 (0.03) | 1 | 1.65 (0.10) | 1 |
| 55-59 | 5.48 (0.06) | 1.18 (0.78-1.77) | 3.63 (0.07) | 1.40 (0.94-2.10) | 1.39 (0.03) | 1.03 (0.63-1.67) | 1.75 (0.11) | 1.20 (0.74-1.95) |
| 60-64 | 5.35 (0.06) | 1.19 (0.78-1.81) | 3.62 (0.06) | 1.24 (0.82-1.90) | 1.39 (0.03) | 0.82 (0.49-1.36) | 1.58 (0.06) | 1.25 (0.76-2.05) |
| 65-69 | 5.40 (0.08) | 1.02 (0.65-1.61) | 3.53 (0.08) | 0.90 (0.56-1.46) | 1.33 (0.03) | 0.91 (0.52-1.58) | 1.51 (0.06) | 0.79 (0.43-1.45) |
| 70-75 | 5.39 (0.10) | 1.21 (0.75-1.94) | 3.60 (0.08) | 1.01 (0.62-1.64) | 1.36 (0.03) | 0.94 (0.54-1.66) | 1.47 (0.08) | 0.94 (0.53-1.70) |
| P value | 0.643 |  | 0.721 |  | 0.501 |  | 0.339 |  |
| **Urban/Rural** |  |  |  |  |  |  |  |  |
| Astana (urban) | 5.85 (0.06) | 1 | 3.70 (0.04) | 1 | 1.29 (0.02) | 1 | 1.68 (0.05) | 1 |
| Akmol (rural) | 5.40 (0.05) | 0.43 (0.32-0.58) | 3.48 (0.04) | 0.67 (0.50-0.89) | 1.45 (0.02) | 0.52 (0.36-0.74) | 1.54 (0.06) | 0.78 (0.55-1.10) |
| P value | <0.001 |  | 0.001 |  | 0.001 |  | 0.036 |  |
| **BMI, (kg/m^2^)** |  |  |  |  |  |  |  |  |
| 18.5-24.9 | 5.46 (0.11) | 1 | 3.41 (0.07) | 1 | 1.57 (0.04) | 1 | 1.16 (0.05) | 1 |
| 25-29.9 | 5.63 (0.06) | 1.71 (1.15-2.56) | 3.61 (0.05) | 1.27 (0.81-1.98) | 1.37 (0.02) | 1.67 (0.95-2.93) | 1.58 (0.07) | 3.34 (1.55-7.21) |
| ≥30 | 5.68 (0.07) | 1.62 (1.09-2.41) | 3.63 (0.05) | 1.39 (0.90-2.16) | 1.28 (0.02) | 2.38 (1.36-4.16) | 1.86 (0.07) | 5.75 (2.70-12.24) |
| P value | 0.291 |  | 0.051 |  | 0.001 |  | 0.001 |  |
| **WHR, obesity** |  |  |  |  |  |  |  |  |
| No | 5.32 (0.07) | 1 | 3.34 (0.06) | 1 | 1.53 (0.03) | 1 | 1.15 (0.03) | 1 |
| Yes | 5.74 (0.05) | 1.97 (1.43-2.71) | 3.70 (0.04) | 2.07 (1.45-2.95) | 1.31 (0.01) | 1.84 (1.18-2.88) | 1.77 (0.05) | 4.81 (2.71-8.55) |
| P value | <0.001 |  | 0.001 |  | 0.001 |  | 0.001 |  |
| **Diabetes** |  |  |  |  |  |  |  |  |
| No | 5.60 (0.04) | 1 | 3.58 (0.03) | 1 | 1.39 (0.01) | 1 | 1.52 (0.04) | 1 |
| Yes | 5.78 (0.14) | 1.39 (0.87-2.21) | 3.66 (0.09) | 1.10 (0.71-1.68) | 1.21 (0.03) | 2.17 (1.36-3.44) | 2.24 (0.16) | 3.09 (2.00-4.77) |
| P value | 0.080 |  | 0.355 |  | 0.001 |  | 0.001 |  |
|  |  |  |  |  |  |  |  |  |
| **Hypertension** |  |  |  |  |  |  |  |  |
| No | 5.69 (0.14) | 1 | 3.54 (0.06) | 1 | 1.41 (0.03) | 1 | 1.49 (0.07) | 1 |
| Yes | 5.64 (0.05) | 1.07 (0.77-1.48) | 3.61 (0.04) | 1.12 (0.80-1.56) | 1.34 (0.02) | 1.01 (0.69-1.50) | 1.68 (0.06) | 1.79 (1.15-2.78) |
| P value | 0.801 |  | 0.341 |  | 0.040 |  | 0.041 |  |
| **Smoking** |  |  |  |  |  |  |  |  |
| Current smoker | 5.84 (0.17) | 1 | 3.71 (0.08) | 1 | 1.29 (0.03) | 1 | 1.68 (0.10) | 1 |
| Past smoker | 5.85 (0.13) | 1.14 (0.71-1.83) | 3.83 (0.13) | 0.70 (0.42-1.17) | 1.35 (0.04) | 0.76 (0.46-1.24) | 1.74 (0.10) | 0.90 (0.51-1.58) |
| Non-smoker | 5.55 (0.06) | 0.95 (0.60-1.51) | 3.51 (0.05) | 0.52 (0.31-0.85) | 1.41 (0.02) | 0.47 (0.29-0.77) | 1.55 (0.06) | 0.65 (0.37-1.14) |
| P value | 0.088 |  | 0.049 |  | 0.024 |  | 0.311 |  |
| **Marital status** |  |  |  |  |  |  |  |  |
| Unmarried | 5.49 (0.14) | 1 | 3.33 (0.10) | 1 | 1.37 (0.07) | 1 | 1.67 (0.27) | 1 |
| Married | 5.61 (0.05) | 1.22 (0.84-1.75) | 3.58 (0.04) | 0.96 (0.68-1.36) | 1.37 (0.02) | 0.67 (0.42-1.07) | 1.61 (0.04) | 1.32 (0.84-2.08) |
| P value | 0.729 |  | 0.749 |  | 0.668 |  | 0.967 |  |
| **Education** |  |  |  |  |  |  |  |  |
| Primary | 5.68 (0.08) | 1 | 3.62 (0.06) | 1 | 1.39 (0.02) | 1 | 1.59 (0.07) | 1 |
| Vocational | 5.48 (0.07) | 0.95 (0.68-1.34) | 3.47 (0.05) | 0.80 (0.56-1.15) | 1.38 (0.03) | 1.22 (0.80-1.86) | 1.65 (0.09) | 1.10 (0.72-1.69) |
| Higher | 5.76 (0.07) | 1.89 (1.29-2.78) | 3.70 (0.06) | 1.24 (0.87-1.76) | 1.32 (0.02) | 1.11 (0.71-1.71) | 1.61 (0.06) | 1.21 (0.79-1.86) |
| P value | 0.022 |  | 0.026 |  | 0.145 |  | 0.729 |  |
| **Ethnicity** |  |  |  |  |  |  |  |  |
| Kazakh | 5.56 (0.07) | 1 | 3.49 (0.04) | 1 | 1.41 (0.02) | 1 | 1.53 (0.04) | 1 |
| Russian | 5.68 (0.08) | 1.35 (0.95-1.92) | 3.68 (0.06) | 1.44 (1.03-2.03) | 1.29 (0.02) | 2.43 (1.62-3.63) | 1.77 (0.12) | 1.36 (0.90-2.05) |
| Other | 5.79 (0.10) | 1.39 (0.91-2.14) | 3.76 (0.08) | 2.11 (1.42-3.14) | 1.30 (0.03) | 1.34 (0.80-2.26) | 1.74 (0.12) | 1.65 (1.03-2.65) |
| P value | 0.039 |  | 0.001 |  | 0.001 |  | 0.023 |  |
| **Car ownership** |  |  |  |  |  |  |  |  |
| No | 5.56 (0.05) | 1 | 3.55 (0.05) | 1 | 1.42 (0.02) | 1 | 1.55 (0.07) | 1 |
| Yes | 5.62 (0.05) | 0.92 (0.68-1.24) | 3.60 (0.04) | 1.22 (0.91-1.65) | 1.34 (0.02) | 1.48 (1.02-2.14) | 1.64 (0.05) | 1.06 (0.74-1.52) |
| P value | 0.596 |  | 0.546 |  | 0.005 |  | 0.443 |  |
| **Deprivation** |  |  |  |  |  |  |  |  |
| High level | 5.55 (0.10) | 1 | 3.53 (0.06) | 1 | 1.38 (0.03) | 1 | 1.46 (0.06) | 1 |
| Intermediate | 5.62 (0.06) | 1.41 (0.97-2.06) | 3.59 (0.06) | 1.19 (0.81-1.75) | 1.39 (0.02) | 0.60 (0.37-0.97) | 1.61 (0.07) | 1.44 (0.89-2.33) |
| Low level | 5.66 (0.06) | 1.53 (1.07-2.19) | 3.62 (0.05) | 1.30 (0.90-1.87) | 1.34 (0.02) | 0.89 (0.58-1.36) | 1.69 (0.07) | 1.44 (0.91-2.28) |
| P value | 0.863 |  | 0.616 |  | 0.372 |  | 0.162 |  |

* Age and sex standardized means of serum lipids; ^1^ Age and sex adjusted prevalence of raised TC ≥ 5 mmol/l (200 mg/dl) or on treatment,

^2^ Age and sex adjusted prevalence of serum lipids: raised LDL ≥ 4.15 mmol/l (160 mg/dl), low HDL < 1.04 mmol/l (40 mg/dl), raised TG ≥ 2.26 mmol/l (200 mg/dl)
